# Evidence of detrimental effects of prenatal alcohol exposure on offspring birthweight and neurodevelopment from a systematic review of quasi-experimental studies

**DOI:** 10.1093/ije/dyz272

**Published:** 2020-01-29

**Authors:** Loubaba Mamluk, Timothy Jones, Sharea Ijaz, Hannah B Edwards, Jelena Savović, Verity Leach, Theresa H M Moore, Stephanie von Hinke, Sarah J Lewis, Jenny L Donovan, Deborah A Lawlor, George Davey Smith, Abigail Fraser, Luisa Zuccolo

**Affiliations:** 1 MRC Integrative Epidemiology Unit, Department of Population Health Sciences, University of Bristol, Bristol, UK; 2 Department of Population Health Sciences, Bristol Medical School, University of Bristol, Bristol, UK; 3 NIHR ARC West, University Hospitals Bristol NHS Foundation Trust, Bristol, UK; 4 Department of Economics, School of Economics, Finance and Management, University of Bristol, Bristol, UK; 5 NIHR Bristol Biomedical Research Centre, University Hospitals Bristol NHS Foundation Trust, University of Bristol, Bristol, UK

**Keywords:** Alcohol, pregnancy, prenatal alcohol exposure, systematic review, quasi-experimental studies, negative control, Mendelian randomization, causal inference, neurodevelopment, FASD

## Abstract

**Background:**

Systematic reviews of prenatal alcohol exposure effects generally only include conventional observational studies. However, estimates from such studies are prone to confounding and other biases.

**Objectives:**

To systematically review the evidence on the effects of prenatal alcohol exposure from randomized controlled trials (RCTs) and observational designs using alternative analytical approaches to improve causal inference.

**Search strategy:**

Medline, Embase, Web of Science, PsychINFO from inception to 21 June 2018. Manual searches of reference lists of retrieved papers.

**Selection criteria:**

RCTs of interventions to stop/reduce drinking in pregnancy and observational studies using alternative analytical methods (quasi-experimental studies e.g. Mendelian randomization and natural experiments, negative control comparisons) to determine the causal effects of prenatal alcohol exposure on pregnancy and longer-term offspring outcomes in human studies.

**Data collection and analysis:**

One reviewer extracted data and another checked extracted data. Risk of bias was assessed using customized risk of bias tools. A narrative synthesis of findings was carried out and a meta-analysis for one outcome.

**Main results:**

Twenty-three studies were included, representing five types of study design, including 1 RCT, 9 Mendelian randomization and 7 natural experiment studies, and reporting on over 30 outcomes. One study design–outcome combination included enough independent results to meta-analyse. Based on evidence from several studies, we found a likely causal detrimental role of prenatal alcohol exposure on cognitive outcomes, and weaker evidence for a role in low birthweight.

**Conclusion:**

None of the included studies was judged to be at low risk of bias in all domains, results should therefore be interpreted with caution.

**Systematic review registration:**

This study is registered with PROSPERO, registration number CRD42015015941

## Introduction

The effects of prenatal alcohol consumption have typically been studied using standard analytical approaches in observational studies.^1^ Systematic reviews have used these types of studies to determine the effects of prenatal alcohol exposure on several outcomes with a wide range and varying definition of alcohol intake including low-moderate to binge drinking. Outcomes such as central auditory disorders in children,^2^ orofacial clefts,^3^ speech and language^4^ and several birth outcomes including low birthweight, preterm birth and small for gestational age^1^^,^^5^^,^^6^ have been investigated. These have led to varying results from systematic reviews: an increased risk of detrimental outcomes at very heavy drinking levels,^1^^,^^2^ inconsistent evidence regarding effects of moderate, heavy, or binge drinking (5+ drinks on any occasion),^3^ inconsistent effects from low-moderate alcohol consumption (up to 83 g/week)^5^ and some evidence that even light prenatal alcohol consumption is associated with harmful birth outcomes (up to 32 g/week).^6^ However, estimates from such studies are prone to the effects of: (i) confounding by socio-demographic characteristics (age, ethnicity, education, socio-economic position) and behavioural factors (smoking and substance use) and (ii) measurement error, namely under-reporting of alcohol intake and/or recall bias. Therefore, the direction and size of any potential causal relationships cannot be determined without bias.

In recent decades, novel analytical approaches have been increasingly applied to data from observational studies in order to improve causal inference when assessing potential effects of prenatal alcohol exposure. These approaches include Mendelian randomization (MR),^7^ family-based designs such as paternal or sibling comparison studies^8^ and natural experiments.^9^ Their respective strengths and limitations are outlined in Box 1.

**Key Messages**

- Systematic reviews of prenatal alcohol exposure effects generally only include conventional observational studies. However, estimates from such studies are prone to confounding and other biases.
- We conducted a comprehensive systematic review of experimental human data and alternative analytical approaches to improve causal inference based on observational data.
- We also developed customized risk of bias tools for Mendelian randomization, natural experiments and parental and sibling comparison, and applied them to studies with these designs.
- Our results showed a likely causal detrimental role of prenatal alcohol exposure on cognitive outcomes, and weaker evidence for a decrease in birthweight, confirming results from conventional observational studies.
- Guidance should continue to advise abstention from alcohol in pregnancy.

**Box 1. Outline of alternative analytical approaches (adapted from** ^10^)

**Table:** 

Causal inference approach	Definition	Biases addressed	Strengths	Limitations
Randomized controlled trial (RCT)	Subjects are randomly allocated to either exposure or control groups with assumption that there is no difference between the two groups except for the intervention they are receiving	Confounding, reverse causality, selection bias, loss-to-follow up bias (using intention-to-treat analysis), measurement error	Gold standard for estimating causal effects. Any effect is very likely to be causal if study has large number and trial is reliably performed	Generalizability may be questionable; impossible or unethical to randomize to certain exposures; can be expensive
Mendelian randomization (MR)	MR is the use of a genetic variant robustly associated with an exposure/risk factor of interest as an instrumental variable to test and estimate the causal effect of that exposure/risk factor with a disease or health related outcome	Confounding by shared genetics and environment, reverse causality, selection bias, measurement errors	Genetic instruments are not subject to confounding from environmental or lifestyle factors, are not influenced by the outcome, do not change over time and are measured with high accuracy	Low power, lack of instrumentation/weak instruments, horizontal pleiotropy, population stratification, inability to assess non-linear associations/dose–response estimation, assortative mating, not specific to the intrauterine period, developmental canalization, subject to dynastic effects bias
Sibling comparison	Compares outcomes when siblings are discordant for an exposure. If causal, then there will evidence of a difference in outcome in relation to discordant exposure levels within sibships	Confounding by genetics and environment, specificity of effect to intrauterine period	Improves causal inference of intrauterine exposures. Controls for familial background and related confounding factors	Assumes a stable family environment; potential for confounding by factors not perfectly shared by siblings; potential for measurement error of exposure; limited power
Natural experiment	Empirical study approach where a population is exposed to an external event or intervention at a specific time point. Associations are then compared with a similar cohort that was not exposed. The assumption is that exposure is caused by quasi-random assignment	Confounding by genetics and environment, reverse causality, specificity of effect to intrauterine period	Can include study settings which would be impractical or unethical to produce by researchers. Allows for long-term effects of the exposure of interest	Selection bias if treatment and control group are not sufficiently comparable, some unobserved confounding may remain, it may not be possible to study non-linear associations/dose–response estimation
Parental comparison	Maternal–child association is compared with paternal–child association for inferring causal effect of intrauterine exposure. If causal, maternal association is stronger than paternal association. Where associations are similar for both parents we assume that they are driven by genetic or postnatal environmental characteristics	Confounding by genetics and environment, specificity of effect to intrauterine period	Improves causal inference of intrauterine effect if exposures are measured in both parents at same time in pregnancy, and non-paternity is taken into account for phenotypic traits	Assumption that paternal exposures share same confounding structure as maternal exposures may not be correct; where parental associations are of similar magnitude this may be due to offsetting paternal pathways rather than shared confounding. Assumes no assortative mating

We conducted a systematic review of human studies that used experimental data [randomized controlled trials (RCTs)] or alternative analytical methods to improve causal inference applied to observational data, in order to determine the causal effects of maternal alcohol consumption in pregnancy on offspring outcomes at birth and later in life. Additionally, as is being recognised elsewhere,^11–13^ it is important in public health and in epidemiology to include work from other disciplines in order to avoid missing important contributions to the literature. We therefore present a co-citation analysis to evaluate whether studies of alcohol in pregnancy carried out in other disciplines, such as health economics, are currently being recognised in public health.

## Methods

### Selection criteria and search strategy

The protocol for this systematic review, carried out using PRISMA guidelines, is available from the PROSPERO systematic review register (registration number CRD42015015941); http://www.crd.york.ac.uk/PROSPERO/display_record.asp? ID=CRD42015015941.

We reported results from prospective observational studies on low-moderate consumption, adopting standard analytical approaches, in a separate manuscript.^6^ Here, we focus on RCTs and studies that used alternative analytical methods to improve causal inference (see Box 1). MR studies that only reported results of geneXenvironment analyses (i.e. stratified by levels of maternal alcohol consumption) were excluded, as these estimates may incur selection bias.^14^

We adopted study specific definitions for all outcomes. Outcomes included the following. (i) Pregnancy outcomes: still birth [pregnancy loss after week 24, miscarriage, gestational length and preterm delivery (<37 weeks gestation)]; hypertensive disorders of pregnancy; gestational diabetes; small for gestational age (SGA, <10th percentile in weight or <−2 standard deviation scores) and birth size [weight (including low birth weight defined as <2500 g), length and head circumference]; low amniotic fluid (oligohydramnios); placenta previa; placental abruption; assisted delivery (including vacuum extraction, forceps delivery, Caesarean section); Apgar score at birth; admission to neonatal unit; congenital malformations. (ii) Features of fetal alcohol spectrum disorder (FASD): childhood growth restriction; cranium size and head circumference; developmental delays; behaviour problems; cognitive impairment and intelligent quotient (IQ); facial malformations.

The databases that were searched included: MEDLINE, PsycINFO, EMBASE on Ovid; the Cochrane Library including CENTRAL (the Cochrane Central Database of Controlled Trials) on Wiley Interscience; and Science Citation Index, Social Science Citation Index, on Web of Science from inception to 21 June 2018 (Supplementary Table 1, available as Supplementary data at *IJE* online). The search was limited to papers in English and excluded letters, animal studies, editorials and conference proceedings without corresponding full-text papers. Investigators tailored searches to each database. The search did not include grey literature and was focused on published medical literature. Additionally, we performed manual searches of the reference lists of: (i) papers included in recent systematic reviews of the effects of prenatal alcohol exposure on the outcomes of interest; and (ii) all recent papers citing those reviews.

Titles and abstracts, and full texts if necessary, were screened independently by two reviewers. Discrepancies were discussed between reviewers and resolved through consensus.

### Data extraction

A custom-built Microsoft Access database was used to extract data. The following information from each study was extracted: title, authors, publication year, country/region, population characteristics (sample size, methods of sampling, age distribution, and ethnicity), study design, measures of exposure, assessment methods for outcomes (including whether this was derived from medical records, obtained via a research interview and the person reporting the outcome e.g. parent, teacher, health professional, researcher or child), model adjustments, and study results. If a study reported more than one result for each outcome, we extracted all of them (e.g. relative to different timing of exposure, model adjustments, etc.). Information from each included paper was extracted by the lead reviewer (L.M.) and subsequently checked for accuracy and completeness by another reviewer (H.B.E.).^15^ There were very few extraction errors and these were resolved through discussion between extractor and checker.

### Data analysis

Odds ratios (OR) and 95% confidence intervals (CI) were derived from count data from individual studies, if they were not reported. Studies were meta-analysed if they used the same analytical approach and estimated the same outcome (e.g. MR analyses of the same genotype–outcome association, discordant siblings’ analyses looking at the same outcome, etc.). The I^2^ statistic was used to determine percentage of variation due to hetrogenity.^16^ Where only two studies were available to meta-analyse, results were not pooled if they were very different from each other.^17^ Alternatively, a narrative summary of the results was given.

### Risk of bias assessment

The Cochrane risk of bias tool was selected to explore risk of bias in eligible randomized control studies.^18^

There are currently no widely accepted risk of bias assessment tools for the alternative observational study designs included in this systematic review (MR, sibling comparison, paternal comparison and natural experiments). We therefore considered the previous work in this area^14^^,^^19^^,^^20^ and adopted key criteria presented in these studies to assess risk of bias. Separate checklists for each of the four study types were developed (Supplementary Tables 3–6, available as Supplementary data at *IJE* online). The checklists mainly focused on the assumptions required for causal inference in these methods (Box 1). Definitions for what would be considered high, medium or low risk of bias for each domain within each separate tool were given. The assessment of each study using the relevant checklist was carried out independently by two reviewers. Conflicts of interest were avoided by making sure any paper whose author was also a reviewer was allocated to another reviewer.

### Co-citation

Co-citation data were collected from Web of Science. These data were analysed using VOSviewer version 1.6.5. Weights/bubble size correspond to the strength of co‐citation. The distant between bubbles corresponds to the number of times that journals are cited together in other journals. The colours correspond to ‘communities’ (clustering) identified by the software, and not pre-specified scientific disciplines.

## Results

**Figure 1:**
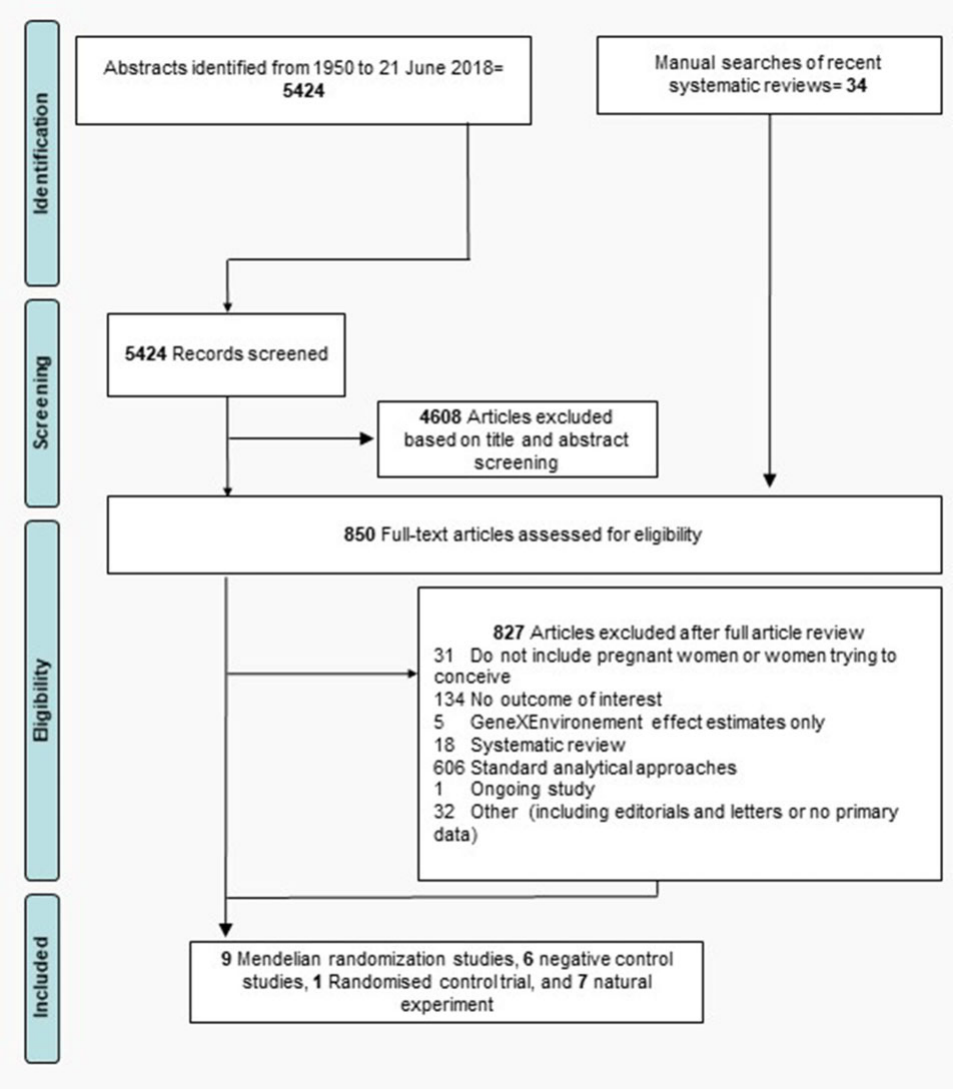
Flowchart of search strategy including primary reasons for article exclusion.

A flowchart of the article review process is shown in Fig. 1. A total of 5424 citation records were identified from searching the four relevant databases. A manual search of recent systematic reviews identified 34 additional articles. After exclusions, 9 MR analyses, 6 negative control studies, 1 RCT and 7 papers based on natural experiments were included, giving a total of 23 studies.

### Risk of bias assessment

Table 1 shows the results of risk of bias assessments. No study was rated low risk of bias in all domains. The RCT was judged at low risk of bias in all except in the blinding domain as participants were not blinded and self-reported their alcohol use. For natural experiment studies the main concerns with regard to validity were the differential trends in outcome, instrument strength and selection bias. For paternal comparison studies potential for differential paternal and maternal confounding and non-paternity were the key threats to validity. In the 2 sibling-comparison studies differential assessment of exposure was the main concern in both studies. All MR studies were rated at moderate risk of having a weak instrument. Further concerns were non-genetic (two studies rated at high risk), genetic confounding and pleiotropy. Because none of the studies are at low risk of bias in all domains for any of the study types, it is not possible to be fully confident in our findings or to predict the direction potential biases could move the results towards. Nevertheless, despite some concerns specific to these study designs, the included studies still provide more robust evidence that is less prone to the type of confounding typically affecting traditional observational epidemiological studies.

**Table 1.** Risk of bias assessment outcomes

Cochrane risk of bias tool for RCTs							
*Study ID*	*Random sequence generation*	*Allocation concealment*	*Blinding of participants/personnel*	*Blinding of outcome assessment*	*Incomplete outcome data*	*Selective reporting*	*Other bias*

Tzilos 2011	Low risk	Low risk	Some concerns (Moderate risk)	Low risk	Low risk	Low risk	Low risk

Risk of bias assessment for Natural experiment studies^a^							
*Study ID*	*Confounding 1—similar populations*	*Confounding 2—differential trends in outcome*	*Confounding 3—population-level cointervention*	*Instrument strength*	*Assessment bias—outcome*	*Selection bias*	

Barreca 2015	Low risk	High risk	Low risk	Unclear risk	Low risk	Moderate risk	
Evans 2016	Moderate risk	Moderate risk	Low risk	Unclear risk	Low risk	High risk	
Fertig 2009	Low risk	Moderate risk	Low risk	Moderate risk	Low risk	High risk	
Nilsson 2017	Low risk	Low risk	Low risk	Moderate risk	Low risk	Low risk	
Zhang 2010	Moderate risk	Moderate risk	Moderate risk	Low risk	Low risk	Moderate risk	
Zhang 2011	Moderate risk	Moderate risk	Moderate risk	Low risk	Low risk	Moderate risk	
Cil 2017	Low risk	Moderate risk	Low risk	Low risk	Low risk	Low risk	

Risk of bias assessment for Parental Comparison studies^b^							
*Study ID*	*Confounding 1—paternal and maternal confounding similar*	*Confounding 2—timing of both parents’ exposure*	*Confounding 3—nonpaternity*	*Confounding 4—paternal effect*	*Assessment bias—exposure*		

McCormack 2018	Moderate risk	Moderate risk	Moderate risk	Moderate risk	Low risk		
Alati 2008	Low risk	Low risk	Moderate risk	Low risk	Low risk		
Alati 2013	High risk	Low risk	Low risk	Low risk	Low risk		
Zuccolo 2016	Moderate risk	Low risk	High risk	Moderate risk	Low risk		

Risk of bias assessment for Sibling Comparison Studies^c^							
*Study ID*	*Confounding 1—birth order*	*Confounding 2— individual level*	*Assessment bias—exposure*	*Assessment bias—outcome*	*Selection Bias*		

D'Onofrio 2007	Moderate risk	Low risk	High risk	Low risk	Low		
Eliertsen 2017	Low risk	Low risk	Moderate risk	Moderate risk	Low risk		

Risk of bias assessment for Mendelian Randomization studies^d^							
*Study ID*	*Weak instrument bias*	*Genetic confounding*	*‘Non-genetic Confounding (e.g. lifestyle factors)*	*Pleiotropy— additional direct effects btw IV & outcome*	*Selection Bias*		

Arfsten 2004	Moderate risk	Moderate risk	High risk	Moderate risk	Moderate risk		
Lewis 2012	Moderate risk	Low risk	Low risk	Low risk	Low risk		
Boyles 2010	Moderate risk	Moderate risk	Moderate risk	Moderate risk	Low risk		
Chevrier 2005	Moderate risk	Moderate risk	Moderate risk	Moderate risk	Low risk		
Stoler 2002	Moderate risk	Moderate risk	High risk	Moderate risk	Low risk		
Viljoen 2001	Moderate risk	Moderate risk	Low risk	Moderate risk	Moderate risk		
Zuccolo 2013	Moderate risk	Low risk	Moderate risk	Low risk	Low risk		
Von Hinke 2014	Moderate risk	Low risk	Low risk	Low risk	Low risk		
Murray 2016	Moderate risk	Moderate risk	Moderate risk	Low risk	Low risk		

a Natural experiment studies. Confounding 1—Similar population: were the populations compared similar with the exception of the naturally randomized exposure? Confounding 2—Differential trends: Has the outcome been changing over time differentially in the populations with and without the naturally randomized exposure?. Confounding 3—Population level cointervention: have there been any other state-level changes coinciding with the natural experiment/intervention? Instrument strength: strength of adherence to law change (natural experiment) or other measures of compliance with instrument presented. Assessment bias—outcome: whether outcome assessment was valid and objective. Selection bias: whether the intervention/natural experiment caused a change in the distribution/characteristics of women getting pregnant, such as to introduce selection bias?

b Parental Comparison studies. Confounding 1—Paternal and maternal confounding similar: assumption that paternal exposures share same confounding structure as maternal exposure. Confounding 2—Timing of both parents exposure: have exposures been measured in both parents at same time in pregnancy? Confounding 3—Nonpaternity: has nonpaternity been taken into account for phenotypic traits? Confounding 4—Paternal effect: is likelihood of a paternal effect also present?

c Sibling Comparison Studies. Confounding 1—Birth order: whether exposed siblings share the same birth order. Confounding 2—Individual level: whether additional intrauterine exposures and post-natal confounders were adjusted. Assessment bias—exposure: whether exposure assessment was free of recall bias. Assessment bias—outcome: whether outcome assessment was valid and objective. Selection bias: Whether there was likely loss to follow up bias for a cohort study.

d Mendelian Randomization studies. Week instrument bias: Strength of association between instrument and exposure F statistic <10 in the same sample. Genetic confounding: whether the study reported test on association between confounders and instrumental variable (IV). Non genetic confounding: Whether the study reports on the distribution of genetic IVs and confounders other than ethnicity (e.g. lifestyle factors). Pleiotropy—additional direct effects btw IV & outcome: whether there is other known effect of genetic variants on outcome or its risk-factors, which is independent of alcohol. Selection bias: Whether the population was homogenous, stratified by ethnicity or adjusted for population stratification.

### Co-citation

**Figure 2:**
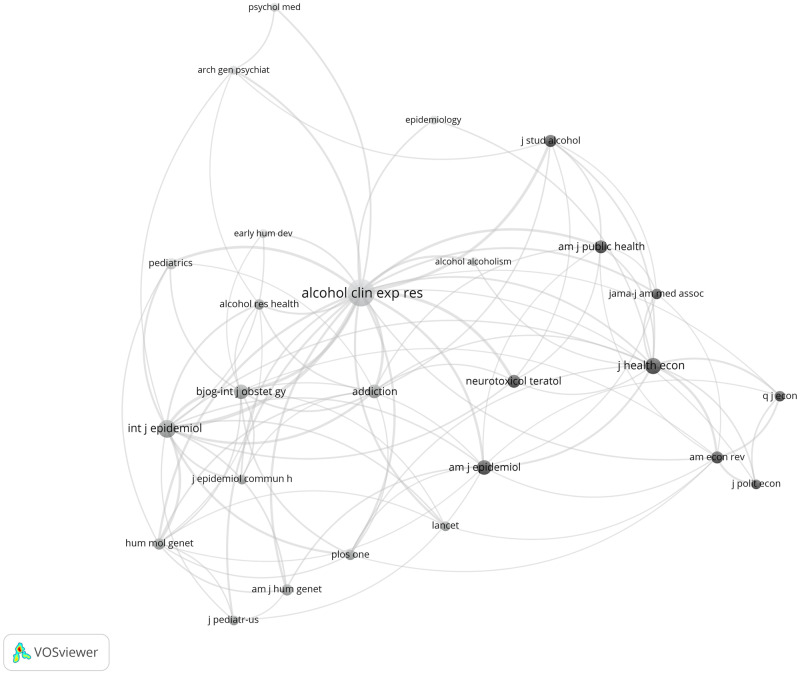
Co-citation of journals. Bubble size corresponds to the magnitude of each journal's citation in the other journals (limit of minimum 8 citations per journal) with a total number of 26 journals. The distance between bubbles corresponds to the number of times with which journals are cited together in other journals. The colours correspond to communities identified by the software (VOS clustering). Produced in VOSviewer version 1.6.5.

Figure 2 illustrates patterns of journal co-citations. It shows four main journal clusters including (health) economics, clinical/alcohol research, genetics and epidemiology. The journal with the highest citation is ‘Alcoholism: Clinical and Experimental Research’. The two other journal disciplines with the highest tendency for co-citation are genetics and epidemiology. The (health) economics cluster has a weaker tendency for co-citation and is the most isolated. The weak cross-disciplinary citation between health economics and other public health/epidemiology/clinical journals could be due to several reasons including differences in the speed of publication as well as in the frequency of citations.

### Mendelian randomization studies

**Figure 3.**
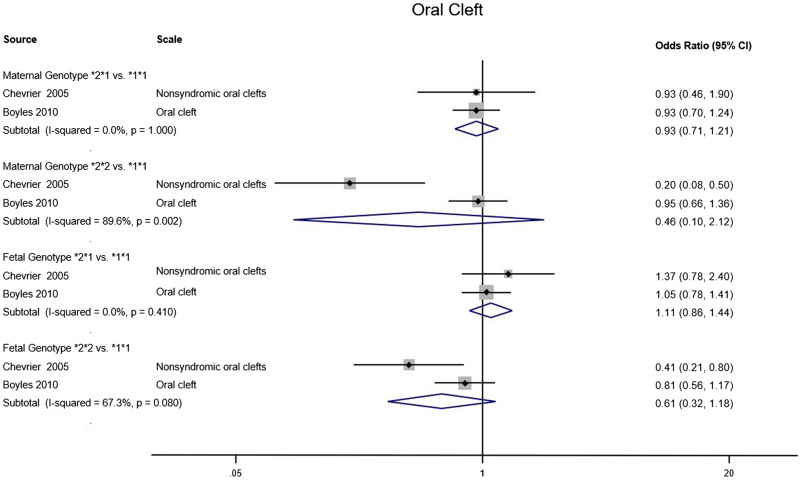
Pooled odds ratios for outcomes of oral cleft in two MR studies.

We identified 9 MR studies examining the effects of prenatal alcohol exposure on pregnancy or offspring outcomes (Table 2). All studies used known variants in alcohol dehydrogenase (ADH) genes in mothers and/or offspring as genetic proxies for the exposure: 5 employed a functional variant in ADH1B,^21–23^^,^^27^^,^^29^ 2 a haplotype in ADH1C,^24^^,^^25^^,^^28^ and 2 a number of ADH variants combined into an allele score^26^^,^^29^ (Table 2). The ADH1B variant is known to alter alcohol metabolic rates^44^ and has been shown to be robustly associated with alcohol consumption levels,^45^ also in pregnant women.^27^ There are two relevant ADH1B polymorphisms, rs1229984 and rs2066702, which define the ADH1B*1, *2 and *3 alleles. The ADH1C haplotype affects alcohol metabolism to a lesser extent^44^ and its effect on alcohol consumption is less clear.^46^Figure 3 shows a meta-analysis over 2 studies^24^^,^^25^ exploring the impact of different maternal and fetal ADH1C alleles on development of infant oral cleft. For three allele comparisons (maternal *2*1 vs*1*1; fetal *2*1 vs*1*1 and fetal *2*2 vs*1*1) the *I*^2^ indicated results in the two studies were reasonably homogeneous, whereas for the maternal *2*2 vs*1*1 comparison, the *I*^2^ showed that the studies were not homogeneous, leading to a much larger overall confidence interval. The meta-analysis provided no evidence for an impact of any of the gene alleles on oral cleft. Two case-control studies examined the risk of oral cleft, comparing faster with slower metabolizers according to ADH1C maternal and fetal genotype. A French study found evidence of lower risk of non-syndromic cleft for ADH1C*2*2 compared with 1*1 homozygotes, but did not report on whether genotype groups differed by alcohol consumption.^24^ The study from Norway found no evidence of association with either offspring cleft risk or maternal alcohol consumption (Table 2).^25^

**Table 2.** Quasi-experimental studies examining the effects of prenatal alcohol exposure (PAE) on pregnancy and childhood outcomes. *3 Denotes rare allele found in sub-Saharan African populations; *2 denotes rare allele found in European ancestry populations; *1 denotes common allele found worldwide

Study (year)	Country	Sample size		Exposure definition (proxy for PAE)	Exposure categories	Age at outcome assessment (child)	Outcomes	Summary of results & conclusions as presented in the paper
Mendelian randomization studies								
Viljoen *et al*. (2001)^21^	South Africa	56 Mother–child pairs 178 Controls Khoisan–Caucasian mixed ancestry	MR	Gene: ADH1B SNP-rs number: rs1229984	*1*1 Slow metabolizers (ref. category) *2* Fast metabolizers *3* Intermediate metabolizers	5–9 years	Fetal alcohol syndrome (FAS)	Fetal genotype: *3* vs *1*1 OR 0.85 (95% CI: 0.·26, 2.72) *2* vs *1*1 OR 0.31 (95% CI: 0·10, 0·90) Maternal genotype: *3* vs *1*1 OR 0.85 (95% CI: 0.26, 2.72) *2* vs *1*1 OR 0.31 (95% CI: 0.10, 0.90)
Stoler *et al*. (2002)^22^	USA	173 White women 85 Black women	MR	Gene: ADH1B SNP-rs number: rs1229984	*1*1 Slow metabolizers (ref. category) *3*1 Intermediate metabolizers	At birth	Blinded physician assessment of a composite trait including: growth restriction, microcephaly or 4 of 6 predefined facial features typical of FAS	Maternal genotype (White women): OR *3*1 vs *1*1: 6.68 (95%CI: 0.89, 49.90) Maternal genotype (Black women): OR *3*1 vs *1*1: 2.62 (95%CI: 0.97, 7.04) Fetal genotype (White children): OR *3*1 vs *1*1: 4.75 (95%CI: 0.35, 64.74) Fetal genotype (Black children): OR *3*1 vs *1*1: 3.75 (95%CI: 1.00, 14.02)
Arfsten *et al*. (2004)^23^	USA	305 African American infants	MR	Gene: ADH1B SNP-rs number: rs1229984	*1*1 Slow metabolizers (ref. category) *3* Intermediate metabolizers	At birth	Birth weight (g) GA (weeks) LBW (<2500g) SGA	Fetal genotype: *3* vs *1*1 3211 vs 3196, *P*=0.83 *3* vs *1*1 39.6 vs 39.4, *P*=0.37 *3* vs *1*1 OR 0.85 (95%CI: 0.30, 2.39) *3* vs *1*1 OR 0.27 (95%CI: 0.06, 1.20)
Chevrier *et al*. (2005)^24^	France	205 Case children + parents (195 fathers and 211 mothers) 115 Control children and 54 mothers	MR	Gene: ADH1C SNPs-rs numbers: rs169342 rs698	Biallelic (2 haplotypes) *1*1 Faster metabolizers (ref. category) *2* Slower metabolizers	−--	Non-syndromic oral clefts	Maternal genotype: *2*1 vs *1*1 OR 0.93 (95% CI: 0.50, 1.90) *2*2 vs *1*1 OR 0.20 (95% CI: 0.10, 0.50) Fetal genotype: *2*1 vs *1*1 OR 1.37 (95% CI: 0.80, 2.40) *2*2 vs *1*1 OR 0.41 (95% CI: 0.20, 0.80)
Boyles *et al*. (2010)^25^	Norway	Mother–child pairs (483 cases 503 controls)	MR	Gene: ADH1C SNP-rs number: rs169342 rs698	Biallelic (2 haplotypes) *1*1 Faster metabolizers (ref. category) *2* Slower metabolizers	At birth	Oral cleft	Maternal genotype: *2*1 vs *1*1 OR 0.93 (95%CI: 0.70, 1.24) *2*2 vs *1*1 OR 0.95 (95%CI: 0.67, 1.36) Fetal genotype: *2*1 vs *1*1 OR 1.05 (95%CI: 0.78, 1.41) *2*2 vs *1*1 OR 0.81 (95%CI: 0.56, 1.17)
Lewis *et al*. (2012)^26^	UK	4 167 Mother–child pairs	MR	Gene: ADH7 SNP-rs number: rs284779 Gene: ADH1B SNP-rs number: rs4147536 Gene: ADH1A SNP-rs number: rs975833 SNP-rs number: rs2866151	Allele score composed of 4 SNPs (unweighted) No information on how it affects metabolic rates	8 years	IQ: Wechsler Intelligence Scale for Children (WISC-III)	Fetal allele score^a^ Beta −1.20 (95% CI: −1.89, −0.52)
Zuccolo *et al*. (2013)^27^	UK	6268 Mother–child pairs for KS2 analysis 4061 pairs for WISC analysis	MR	Gene: ADH1B SNP-rs number: rs1229984	Biallelic SNP *1*1 Slow metabolizers (ref. category) *2* Fast metabolizers	8 years	Total IQ: Wechsler Intelligence Scale for Children (WISC)	Maternal genotype:*2* vs *1*1 MD −0.01 (95% CI: -2·8, 2·7)
						11 years	Academic achievement: Key Stage 2 scores	Maternal genotype: *2* vs *1*1 MD 1.7 (95% CI: 0.4, 3.0) Fetal genotype: *2* vs *1*1 MD 0.68 (95% CI: −0.24, 1.60)
von Hinke Kessler Scholder *et al*. (2014)^28^	UK	Mother–child pairs KS1: 3255 KS2: 3067 KS3: 2812 KS4: 3138	MR	Gene: ADH1B SNP-rs number: rs1229984	Biallelic SNP *1*1 Slow metabolizers (ref. category) *2* Fast metabolisers	7–16 years	Academic achievement: Key Stage 1 scores (age 7) Key Stage 2 scores (age 11) Key Stage 3 scores (age 14) Key Stage 4 scores (age 16)	Maternal genotype: *2* vs *1*1 MD 0.198 (95% CI: 0.05, 0.35) Fetal genotype: *2* vs *1*1 MD −0.082 (95% CI: -0.24, 0.07) Maternal genotype: *2* vs *1*1 MD 0.239 (95% CI: 0.08, 0.40) Fetal genotype: *2* vs *1*1 MD −0.122 (95% CI: −0.30, 0.06) Maternal genotype: *2* vs *1*1 MD 0.192 (95% CI: 0.03, 0.36) Fetal genotype: *2* vs *1*1 MD −0.103 (95% CI: −0.28, 0.07) Maternal genotype: *2* vs *1*1 MD 0.25 (95% CI: 0.10, 0.40) Fetal genotype: *2* vs *1*1 MD −0.075 (95% CI: −0.22, 0.07)
Murray *et al*. (2016)^29^	UK	Maternal genotype analysis: 3114 mother–child pairs	MR	Gene: ADH1B SNP-rs number: rs1229984	*1*1 Slow metabolizers (ref. category) *2* Fast metabolizers	4–13 years	Child’s conduct problem trajectories aged 4–13 based on Strengths and Difficulties Questionnaire (SDQ), categorized as: low-risk (ref) early-onset-persistent	Maternal genotype: OR 1.20 (0.60, 2.44) for early-onset-persistent conduct problems vs low-risk^b^
Sibling control studies								
D’Onofrio *et al*. (2007)^30^	USA	8621	Sibling comparison	Siblings discordant for PAE	Moderate alcohol exposure vs none	4–11 years	Conduct problems Attention/impulsivity problems	Moderate vs no alcohol intake: MD 0.05 SE: 0.02 Moderate vs no alcohol intake: MD 0.03 SE: 0.02
Eilertsen *et al*. (2017)^31^	Norway	34 283	Sibling comparison	Siblings discordant for PAE	Not available	5 years	Attention-deficit hyperactivity disorder (ADHD) Scales: the revised Conner’s Parent Rating Scale (CPRS-R) Child Behaviour Checklist (CBCL)	(β= 0.017, 95% CI: 0.005, 0.030) (β= 0.011, 95% CI: -0.002, 0.024)
Parental control studies								
Alati *et al*. (2008)^32^	UK	4332	Maternal–paternal comparison	Mother and partner alcohol intake	First trimester (regular use): never <1 drinks /week 1–6 drinks/week 7+ drinks/week Binge drinking: 1–4 drinks/occasion 5–10 drinks/occasion 10+ drinks/occasion	8 years	IQ: Wechsler Intelligence Scale for Children (WISC)	1st trimester regular alcohol use (pdiff = *0·43)* MD per increase in maternal category: 0.03 (−0.58, 0.65) MD per increase in paternal category: 0.40 (−0.01, 0.82) Binge drinking; (*P*diff =0.38*)* MD per increase in maternal category: −0.45 (−1.32, 0.43) MD per increase in paternal category: 0.10 (−0.36, 0.56)
Alati (2013)^33^	UK	7062	Maternal–paternal comparison	Mother and partner alcohol intake	First trimester (regular use): never <1 drinks/week 1–6 drinks/week 7+ drinks/week Binge drinking: 1–4 drinks/occasion 5–10 drinks /occasion 10+ drinks/occasion	11 years	Academic achievement: Key Stage 2 scores (standardized)	1st trimester regular Alcohol use (*P*diff = *0·406)* MD per increase in maternal alcohol category: Adjusted^c^ MD: 0.10 (-0.17, 0·37) MD per increase in paternal alcohol category: 0.25 (0.07, 0·43), (pdiff 0.41) Binge drinking; (*P*diff <0.0001*)* MD per increase in maternal category: −0.68 (−1.03, −0.33) MD per increase in paternal category: 0.27 (0.07, 0.46)
Zuccolo *et al*. (2016)^34^	Norway	46 178	Maternal–paternal comparison	Mother and partner alcohol intake	Non-drinker (ref) <1 unit/week 1–2 units/week 3–4 units/week 5+ units/week	At birth and at 3 months post-partum	Head circumference Microcephaly	At birth- Fully and mutually adjusted model^c^ <1unit- Mother: mean difference (SD) 0.00 (95% CI: −0.02, 0·02) Father: mean difference (SD) −0.00 (95% CI: −0.05, 0.04) 1–2 units- Mother: mean difference (SD) −0.02 (95% CI: −0.05, 0.01) Father: mean difference (SD) 0.01 (95% CI: -0.03, 0·05) 3–4 units- Mother: mean difference (SD) 0.06 (95% CI: 0.02, 0.11) Father: mean difference (SD) 0.01 (95% CI: −0.03, 0.05) 5+ units- Mother: mean difference (SD) 0.01 (95% CI: −0.04, 0.06) Father: mean difference (SD) −0.01 (95% CI: −0.04, 0.03) At 3 months post-partum: fully and mutually adjusted model^d^ < 1unit- Mother: mean difference (SD) 0.02 (95% CI: −0.00, 0.05) Father: mean difference (SD) -0.02 (95% CI: -0.08, 0.04) 1–2 units- Mother: mean difference (SD) −0.02 (95% CI: −0.05, 0.02) Father: mean difference (SD) −0.03 (95% CI: −0.08, 0.02) 3–4 units- Mother: mean difference (SD) 0.04 (95% CI: −0.02, 0.10) Father: mean difference (SD) −0.04 (95% CI: −0.09, 0.01) 5+ units- Mother: mean difference (SD) 0.05 (95% CI: −0.02, 0.12) Father: mean difference (SD) −0.05 (95% CI: −0.10, 0.00) At birth: fully and mutually adjusted model^e^ <1 unit- Mother: OR 0.68 (95% CI: 0.50, 0.94) Father: OR 1.00 (95% CI: 0.53, 1.88) 1–2 units- Mother: OR 1.13 (95% CI: 0.81, 1.59) Father: OR 1.11 (95% CI: 0.66, 1.87) 3–4 units- Mother: OR 0.97 (95% CI: 0.57, 1.68) Father: OR 1.21 (95% CI: 0.72, 2·06) 5+ units- Mother: OR 1.22 (95% CI: 0.68, 2.20) Father: OR 1.36 (95% CI: 0.81, 2.28) At 3 months post-partum: fully and mutually adjusted model^f^ <1 unit- Mother: OR 0.82 (95% CI: 0.66, 1.02) Father: OR 1.25 (95% CI: 0.79, 1.95) 1–2 units- Mother: OR 0.82 (95% CI: 0.62, 1.09) Father: OR 1.16 (95% CI: 0.79, 1.71) 3–4 units- Mother: OR 1.08 (95% CI: 0.73, 1.59) Father: OR 1.38 (95% CI: 0.94, 2.03) 5+ units- Mother: OR 0.82 (95% CI: 0.49, 1.39) Father: OR 1.33 (95% CI: 0.90, 1.95)
McCormack *et al*. (2018)^35^	Australia	2030	Maternal–paternal comparison	Mother and partner alcohol intake	First 6 weeks: Abstinent Low Moderate Binge Heavy Second 6 weeks Abstinence Low Trimester 2: Abstinence Low Trimester 3 Abstinence Low	12 months	Infant cognitive development (Bayley Scales for Infant Development, third edition)	Maternal alcohol use (compared with abstinence): Trimester 1: first 6 weeks Low: (β −0.45, SE 0.86) Moderate: (β 1.·35, SE 1.61) Binge: (β −0.90, SE 0.96) Heavy: (β −0.13, SE 1.02) Trimester 1: second 6 weeks Low: (β 0.54, SE 0.86) Trimester 2 Low: (β 2.11, SE 0.77) Trimester 3 Low: (β 1·60, SE 0.77) Partners alcohol use (compared with abstinence): Low: (β 2.42, SE 1.55) Moderate: (β 0.67, S.E 1.81) Heavy: (β 2.19, SE 1.98) Binge: (β 2.00, SE 1.58)
Natural experiments								
Fertig and Watson (2009)^36^	USA	All women 16 165 747 Native White women 11 426 203 Native Black women 3 032 108		Changes in minimum legal drinking age (MLDA)	Lower (18 years) vs higher (19–21 years) MLDA	At birth	Low birth weight (<2500g) Preterm birth (<37 weeks) Congenital anomalies	MLDA of 18 main effect; -0.17% (SE 0·07)^g^ MLDA of 18 x mother ≤17 years of age interaction; 0.50% (SE 0.18)^g^ MLDA of 18 x mother 18–20 years of age interaction; 0.26% (SE 0.10)^g^ MLDA of 18 main effect; −0.35% (SE 0.09)^g^ MLDA of 18 x mother ≤17 years of age interaction; 0.86% (SE 0.28)^g^ MLDA of 18 x mother 18–20 years of age interaction; 0.26% (SE 0.12)^g^ MLDA of 18 main effect; -0.18% (SE 0.12)^g^ MLDA of 18 x mother ≤17 years of age interaction; −0.04% (SE 0.05)^g^ MLDA of 18 x mother 18–20 years of age interaction; −0.03% (SE 0.02)^g^
Zhang (2010)^37^	USA	Infants with birthweight 71 501 237 Infants with APGAR scores 55 054 916		Raising alcohol taxes	Higher vs lower alcohol tax	At birth	Birthweight (g) Low birthweight (<2 500 g) Extremely low birthweight (<1 500 g) Low APGAR scores (<7)	Beer tax (β 0.931, SE 0.003) Wine tax (β 0.340, SE 0.006) Liquor tax (β 0·072, SE 0.027) Beer tax (β -0.023, SE 0.001) Wine tax (β -0·006, SE 0.0004) Liquor tax (β -0.001, SE 0·0001) Beer tax (β −0·002, SE 0·0004) Wine tax (β −0·002, SE 0·001) Liquor tax (β −0·001, SE 0·001) Beer tax (β -0·0002, SE 0·000001) Wine tax (β 0·0002, SE 0·0002) Liquor tax (β −0·0001, SE 0·0000)
Zhang and Caine (2011)^38^	USA	All women (<21 years) 26 743 White women (<21 years) 16 596 Black women (<21 yrs) 11 147		State-specific MLDA when woman is 14 years as proxy of alcohol availability	Effects of different MLDA (18–2121 years), when woman is 14 (e.g. proxying for different alcohol availability)	At Birth	Low birthweight (<2 500 g) Low APGAR scores (<7) Preterm birth (<37 weeks)	MLDA of 18 (vs higher); 0·14% (*P*< 0·0001)^h^ MLDA of 19 (vs 18); −0·16% (*P*=0.002)^h^ MLDA of 20 (vs 18); −0·05% (*P*=0·217)^h^ MLDA of 21 (vs 18); −0·24% (*P*<0·0001)^h^ MLDA of 18 (vs higher); 1·12% (*P*< 0·0001)^h^ MLDA of 19 (vs 18); −1.80% (*P*<0·0001)^h^ MLDA of 20 (vs 18); −1.03% (*P*<0·0001)^h^ MLDA of 21 (vs 18); −1.82% (*P*<0·0001)^h^ MLDA of 18 (vs higher); 0·02% (*P* = 0.051)^i^ MLDA of 19 (vs 18); −0.02% (*P* =0·051)^i^ MLDA of 20 (vs 18); 0.01% (*P* =0·215)^i^ MLDA of 21 (vs 18); −0.04% (*P*=0·0002)^i^
Barreca and Page (2015)^39^	USA	14–17 years at conception 3 314 000 18–20 years at conception 6 287 000 21–24 years at conception 10 178 000		Differences in MLDA	Lower (18 years) vs higher (19–21) MLDA	At birth	Low birthweight (<2500 g) Preterm birth (<37 weeks) Low Apgar score (<7) Congenital anomaly Female	MLDA of 18 main effect; -0.19% (SE 0.08)^j^ MLDA of 18 x mother 14–17 years of age interaction; −0.02% (SE 0.07)^j^ MLDA of 18 x mother 18–20 years of age interaction; 0.10% (SE 0.05)^j^ Mean of outcome: 7.5 MLDA of 18 main effect; −0.04% (SE 0.10)^j^ MLDA of 18 x mother 14–17 years of age interaction; 0.05% (SE 0.12)^j^ MLDA of 18 x mother 18–20 years of age interaction; 0·04% (SE 0.09)^j^ Mean of outcome: 10.7 MLDA of 18 main effect; 0.39% (SE 0.32)^j^ MLDA of 18 x mother 14–17 years of age interaction; 0.46% (SE 0.53)^j^ MLDA of 18 x mother 18–20 years of age interaction; 0.19% (SE 0.26)^j^ Mean of outcome: 901.4 MLDA of 18 main effect; −0.28% (SE 0.20)^j^ MLDA of 18 x mother 14–17 years of age interaction; −0.002% (SE 0.06)^j^ MLDA of 18 x mother 18–20 years of age interaction; 0.04% (SE 0.03)^j^ Mean of outcome: 8.0 MLDA of 18 main effect; −0.01% (SE 0.12)^j^ MLDA of 18 x mother 14–17 years of age interaction; 0.01% (SE 0.10)^j^ MLDA of 18 x mother 18–20 years of age interaction; 0.18% (SE 0.07)^j^ Mean of outcome: 48.8
Evans *et al*. (2016)^40^	USA	1 704 191 (Education sample) 985 118 (Obesity/height sample)		Effect of state alcohol prohibitions	States with alcohol prohibitions vs states without prohibitions	*In utero*, 8, and 10 years of exposure to state alcohol prohibitions	Adult education attainment Height	Exposure before 8 years of age 0·04 (SE 0·01) per year Exposure before 10 years of age 0.05 (SE 0.01) per year Exposure *in utero* 0.08 (SE 0.05) Exposure before 8 years of age 0.·0001 (SE 0.0001) per year Exposure before 10 years of age 0.0002 (SE 0.0001) per year Exposure *in utero* 0.0003 (SE 0·0005)
Cil (2017)^41^	USA	Birth weight: 60 914 264 Pre-term: 53 276 541 FAS: 28 371 025 APGAR: 48 291 613		Effect of point-of-sale warnings about risks of drinking during pregnancy	States with warnings vs states without warnings (including pre-/post-intervention within states)	At birth	Low birth weight (<2500 g) Very low birth weight (<1500 g) Pre-term (<37 weeks) Very pre-term (<32 weeks) FAS Low APGAR (<7)	−0.115% (SE 0.082) decreased odds of low birth weight (*P* > 0.1) −0.047% (SE 0.023) decreased odds of very low birth weight (*P* < 0.05) −0.065% (SE 0.14) decreased odds of pre-term birth (*P* > 0.1) −0.052% (SE 0.029) decreased odds of very pre-term birth (*P* >0.1) −0.003% (SE 0.002) decreased odds of FAS (*P* > 0.1) −0.014% (SE 0.047) decreased odds of low APGAR (*P* > 0.1)
Nilsson (2017)^42^	Sweden	353 742		Relaxing the regulation of alcohol sales	Children born in counties with relaxed regulation on alcohol sales vs those born in counties with stronger regulation on alcohol sales (Exposed from first half of pregnancy)	Mean age 32 years	Earning, education and welfare dependency rate	Exposed children had: A reduction in years of schooling −0.31 (SE 0.09) years, males −0.52 (SE 0.17) years, females -0.21 (SE 0.12) Were less likely to complete high school −0.63 (0.02), males −0.1(SE 0.02), females -0.03 (SE 0.03) Had lower (log) earnings −0.24 (SE 0.09), males −0.24 (SE 0.11), females -0.17 (0.14) An increased risk of no labour income 0.07 (SE 0.03), males 0.08 (SE 0.02), females 0.06 (SE 0.04) A higher proportion were on welfare 0.04 (0.01), males 0.05 (SE 0.02), females 0.03 (SE 0.01)
Randomized controlled trial								
Tzilos *et al*. (2011)^43^	USA	Intervention group 27 Control group 23		Computer-delivered brief intervention for reduced prenatal alcohol use (33 days)	Children born to mothers receiving intervention on prenatal alcohol use vs to mothers receiving standard care	At birth	Birthweight	Intervention group: mean= 3189.6, SD=328.0 Control group: mean= 2965.3, SD= 387.7

LBW, low birth weight; ELBW, extremely low birthweight; GA, gestational age; APGAR, appearance, pulse, grimace, activity, respiration; MLDA, minimum legal drinking age; NICU, neonatal intensive care unit; OR, odds ratio; CI, onfidence interval; MD, mean difference; SD, standard deviation; SGA, small for gestational age; IQ: intelligence quotient; RR, relative risk.

a unweighted allele score composed of the four fetal SNPs.

b Adjustments: mother’s ancestry principle components from Genome wide association studies** (**GWAS) analysis.

c Adjustments: sex, other parent’s alcohol consumption, maternal age, parity, socio-economic position, ethnicity, and, maternal and paternal education and smoking.

d Test for trend maternal and paternal alcohol intake in full model: *P* = 0.267 and *P* = 0.201.

e Test for trend maternal and paternal alcohol intake in full model: *P* = 0.390 and *P* = 0.124.

f Test for trend maternal and paternal alcohol intake in full model: *P* = 0.545 and *P* = 0.056.

g Test for trend maternal and paternal alcohol intake in full model: *P*= 0.178 and *P* = 0.090.

h Adjustments: state fixed effects, year–month fixed effects, maternal age fixed effects, state-specific time trends and birth characteristic controls.

i Adjustments: state fixed effects, year fixed effects, mother’s education, age, marital status, smoking during pregnancy, real income per capita and real beer taxes (federal plus state level).

j Adjustments: state fixed effects, year-by-month fixed effects, age fixed effects, state-specific trends, age-by-year fixed effects, state-by-age fixed effects and state-by-year fixed effects.

#### Pregnancy outcomes

A study of African American infants found no strong evidence of association between infant ADH1B genotype and measures of birth size and gestational age, but did not report levels of maternal alcohol use by genotype^23^ (Table 2).

#### Features of FASD

The US-based study by Stoler *et al*.^22^ found some evidence of higher odds of a FASD-like construct in offspring carrying the ADH1B*3 allele compared with *1*1 homozygotes (Table 2). The latter metabolize alcohol more slowly and were also reported to have been exposed to lower levels of alcohol in pregnancy. The same direction of effect was observed comparing offspring of mothers carrying ADH1B*3, and the evidence was stronger for those of black ethnicity.^22^ Another study on fetal alcohol syndrome (FAS), from South Africa, found evidence of lower risk comparing carriers of maternal (or fetal) (fast metabolizing and lower alcohol intake) ADH1B*2 with ADH1B*1*1 homozygotes (slower metabolizers and higher intake), and little evidence of an effect of ADH1B*3 on FAS, in a mixed-ancestry South African population (Table 2).^21^ This study did not report on genotype–alcohol use association.

#### Other outcomes

The four most recent (and by far the largest) MR studies reported on cognitive and behavioural childhood outcomes in the same UK-based cohort (Table 2).^26–29^ Two used multiple offspring ADH variants known to be expressed in fetal life. One of these found evidence of association with IQ at 8 years old, but not when using the maternal allele score;^26^ the effects were stronger for children of mothers reporting some alcohol consumption, but there was no evidence of association between the allele score and maternal alcohol use per se. The other study did not find an association between maternal genotype ADH1B*2* and an increased risk of children having early-onset-persistent behavioural problems, however this may be due to lack of statistical power (Table 2).^29^

The other two studies both used the functional ADH1B variant, and found some evidence that the offspring of mothers genetically predisposed to consuming less alcohol had better academic performance at ages 7, 11, 14 and 16, but no association between offspring genotype and their educational outcomes,^28^ nor was there evidence for an effect of genotype on IQ.^27^ Both studies reported lower alcohol consumption in mothers carrying the rare ADH1B*2 allele compared with the ADH1B*1*1 homozygotes.

### Sibling comparison studies

Two sibling-comparison studies compared behavioural outcomes in siblings differentially exposed to alcohol *in utero* (Table 2).

#### Features of FASD

The study from the USA examined externalizing problems (measured through the Behaviour Problem Index) at ages 4–11 and found evidence that siblings exposed to moderate levels of prenatal alcohol had higher rates of conduct problems compared with their unexposed siblings, however there was no evidence of differences in attention or impulsivity problems.^30^ The more recent study from Norway compared differentially exposed siblings in terms of their attention-deficit hyperactivity disorder (ADHD) at 5 years of age.^31^ Results differed slightly depending on the ADHD scale used, with evidence of increased prenatal alcohol exposure being associated with higher ADHD levels according to the revised Conner’s Parent Rating Scale, but less strong evidence for the Child Behaviour Checklist.^31^

### Parental comparison studies

Four maternal–paternal comparison studies met our inclusion criteria. These investigated the effects of prenatal alcohol exposure on neurocognitive domains in offspring: childhood educational achievement,^33^ IQ,^32^ cognitive development^35^ and head circumference^34^ (Table 2).

#### Features of FASD

Two reports from the same UK-based study found no evidence of association between regular maternal alcohol use in pregnancy and either school results at 11^33^ or IQ at 8 years of age.^32^ One of the studies did find some evidence that increased levels of maternal binge drinking in pregnancy (consuming 32+g alcohol/occasion) were associated with decreased school results at age 11 years, whereas paternal exposure was associated with improved school results.^33^ The other report did not find the same level of evidence to support an association of prenatal binge drinking with offspring IQ at age 8 years.^32^ In a large Norwegian cohort, there was no evidence of association between maternal or paternal alcohol use during or before pregnancy and head circumference at birth or 3 months.^34^ In the same study, odds of microcephaly increased with higher paternal but not maternal alcohol consumption prior to pregnancy and in the first trimester.^34^ A recent Australian study showed no consistent evidence of association between maternal alcohol use in different trimesters of gestation and cognitive function in children aged 1 year (Bayley Scales of Infant Development), and even scanter evidence for partner alcohol intake.^35^

### Natural experiments

Seven reports analysed data from natural experiments involving changes in government laws that effected the availability or affordability of alcohol,^36–42^ or required point-of-sale warnings about the risks of drinking alcohol during pregnancy^41^ (Table 2).

#### Pregnancy outcomes

Three US-based studies used reductions in the minimum legal drinking age (MLDA) to proxy for prenatal alcohol exposure, under the assumption that a lower MLDA would increase alcohol availability to young women^36^^,^^38^^,^^39^ (Table 2). The studies by Fertig and Watson^36^ and Barreca and Page^39^ were based on US-wide birth data and estimated the association between MLDA and low birthweight (<2500 g), preterm delivery (<37 weeks) and congenital anomalies, with the latter additionally examining Apgar scores. Both used a triple difference approach (Supplementary Material, available as Supplementary data at *IJE* online) and substantially the same data, although the latter study ran additional analyses with more covariates and interaction terms to check the robustness of the model to some of its assumptions. When running similar age-specific analyses, the second study replicated the first study’s results of an increase in both preterm deliveries and low birthweight corresponding to a lowering of MLDA, more marked for babies conceived to younger (<18 year old) compared with older (18–20 year old) women.^36^^,^^39^ In more fully adjusted analyses, the negative association with birthweight was still found to be robust for younger mothers (<18 years). However, no consistent evidence of association was found for other age groups in the main effects analyses, or for other adverse fetal outcomes including gestational age, congenital abnormalities and Apgar score.^39^ Neither study reported data on actual population-level alcohol use. The third study, by Zhang and Caine (2011),^38^ investigated the same outcomes (low birthweight, preterm delivery and Apgar scores) in relation to a State’s MLDA at the time a woman is 14 years old. The difference with respect to the two previous studies was that the ‘exposed’ status is assigned based on MLDA at the time the women are 14 years, regardless of what it is when she is older and pregnant. The authors hypothesize that the drinking environment at age 14 sets a woman’s future ‘drinking propensity’ including binge drinking behaviour, but no data were reported to confirm this. The estimates were derived from difference-in-difference specifications, but with additional controls for State-specific effects. The authors presented evidence that women who lived in a State where the MLDA was 18 years at the time they themselves were 14 years, compared with those in States with higher MLDA, had higher chances of giving birth to low birthweight babies with lower Apgar scores, but no association with prematurity.^38^

A fourth paper examined the effect of within-State changes in alcohol taxation in the US and within-State variation in birthweight and Apgar scores^37^ (Table 2). The authors found evidence that increases in alcohol taxes are associated with increases in birthweight and Apgar scores. The authors also tried to validate their assumptions that changes in taxation are a valid proxy for alcohol consumption and therefore prenatal alcohol exposure, by regressing several alcohol drinking variables from a federal behavioural survey on alcohol taxation. They found some evidence of reduced binge drinking behaviour among pregnant women, corresponding to increases in alcohol taxes, however no evidence that the quantity consumed was sensitive to alcohol pricing.^37^

Another US-based study explored the impact of State laws requiring point-of-sale warnings about the risks of drinking alcohol during pregnancy on outcomes including birthweight, pre-term birth, FAS and Apgar scores.^41^ There was evidence that the warnings reduced the chances of very low birth weight babies (<1500 g), but no evidence of association with the other outcomes. The authors validated their assumption that alcohol warning signs would reduce prenatal alcohol exposure by regressing several alcohol drinking variables on whether the State prescribed health warnings or not, using both individual birth and national survey data. They found that adoption of the law was associated with a reduction in alcohol consumption and binge drinking among pregnant women.

#### Features of FASD

Two studies looked at long-term offspring outcomes (Table 2). Based on data from World War II US enlistees, the first study used different timings of prohibition implementation in different States to proxy for reduced likelihood of prenatal alcohol exposure as a result of reduced availability to women, and examined attained education and height in adult offspring.^40^ The authors report an increase in years of education associated with the introduction of prohibition, but no evidence of an effect on height. However, there were no estimates of actual alcohol consumption in States introducing prohibition.^40^

A Swedish study compared earnings, education and welfare dependency rates in children born in counties that did and did not relax the regulation of alcohol sales in 1967.^42^ The relaxation of alcohol policy, used as a proxy for increased prenatal alcohol exposure, was shown to be related to reduced earnings, years of schooling and high school completion rates, as well as to a higher proportion of individuals on welfare.^42^ The author reported some evidence of increased consumption of alcohol for the counties during the period where the more liberal policy applied, but no results specifically for pregnant women.

### Randomized controlled trial

We included one RCT^43^ feasibility study with a small sample size (control group 23 women, intervention group 27 women; Table 2).

#### Pregnancy outcomes

In the RCT feasibility study, 50 pregnant women who screened positive for risky drinking were randomized: 27 pregnant women in the intervention group received a 20-min computer-based, self-administered program intended to motivate them to reduce their drinking, whereas 23 pregnant women in the control group received a questionnaire about television preferences. Follow-up after 1 month (average 33 days) showed no difference in alcohol use between the intervention and control groups but some evidence of higher birthweight for infants born to women in the intervention group compared with the control group. As there was no strong evidence of a difference in alcohol consumption between the randomized groups this does not support any causal effect of alcohol on birthweight but may suggest bias in the RCT, some pathways (other than change in alcohol) from the intervention to birthweight that might counter any effect of alcohol and/or too little power to detect effects on alcohol robustly.

## Discussion

### Summary of the evidence

Our systematic review of the literature found a limited number of studies addressing the effects of prenatal alcohol exposure using experimental designs or alternative analytical strategies to improve causal inference in observational studies, which we described in narrative format. Twenty-three reports were included, representing five types of study design, with MR and natural experiments the most common designs (9 and 7 studies, respectively). Cognitive outcomes were the most commonly reported (by 9 studies), followed by birthweight (7 studies). The overall picture that emerges from this review is that moderately strong evidence exists for detrimental effects of prenatal alcohol exposure on cognitive outcomes (Table 3). For cognitive outcomes and birth weight outcomes, we found the highest degree of consistency across study types (MR,^26–28^ parental comparisons^33^ and natural experiments exploiting different policy changes^40^^,^^41^) as well as with the direction of association predominantly reported in conventional epidemiological studies.^47^^,^^48^ Based on natural experiments^36–39^ and one feasibility RCT,^43^ some evidence was also found for reduced birthweight following higher prenatal alcohol exposure (Table 3), in line with recent reviews^6^ and pooled analyses of observational studies.^49^

**Table 3.** Summary of direction of association of prenatal alcohol exposure with selected outcomes (cognitive/brain development and birthweight), in the context of expected and observed differences in prenatal alcohol exposure in each study

Outcome	Study	Direction	Direction	
		PAE→outcome	Exposure proxy→PAE	
			Expected	Observed
Cognition, brain development				
	Lewis *et al*. (2012)^26^	↓	NA (Not Applicable)	NA
	Zuccolo *et al*. (2013)^27^	↑	↓	↓
	von Hinke Kessler Scholder *et al*. (2014)^28^	↑	↓	↓
	Zuccolo *et al*. (2016)^34^	↔	↑	↑
	McCormack *et al*. (2018)^35^	↔	↑	↑
	Alati *et al*. (2008)^32^	↔	↑	↑
	Alati *et al*. (2013)^33^	↓	↑	↑
	Nilsson (2017)^42^	↓	↑	NA
	Evans *et al*. (2016)^40^	↑	↓	NA
Birthweight				
	Arfsten *et al*. (2004)^23^	↔	NA	NA
	Fertig and Watson (2009)^36^	↓	↑	NA
	Barreca and Page (2015)^39^	↓	↑	NA
	Zhang and Caine (2011)^38^	↓	↑	NA
	Zhang (2010)^37^	↑	↓	↓
	Cil (2017)^41^	↓	↑	↑
	Tzilos *et al*. (2011)^43^	↑	↓	↔

Only one outcome-study design combination had more than one result that could be combined into a meta-analysis. For the rest, we described results in narrative format. We also developed and deployed customized risk of bias (RoB) assessment tools for the different types of study design. None of the studies scored ‘low’ RoB in all domains, therefore we recommend caution in interpreting the results of any one study as ‘causal’, since it is impossible to predict the overall direction of bias affecting each result.

Results of our co-citation analysis showed that the field of (health) economics is relatively isolated compared with the other clusters. It also shows a limited number of studies in public health. This shows that the findings published in health economics journals are not well recognised in the fields of epidemiology and public health, although the evidence they contribute should be considered alongside that from more traditional epidemiological studies when updating public health guidance on alcohol use, as evidenced by our reviewing efforts.

### Strengths and limitations of alternative study designs

An extensive literature exists exploring the strengths and limitations of the observational study designs and analytical strategies^19^ included in this review, especially when applied to the study of intergenerational effects such as here.^50^^,^^51^ In theory, all study types attempt to minimize confounding by shared genetic and environmental factors by design, all but MR and some of the natural experiments address the specificity of the effect to the intrauterine period (i.e. not confounded by postnatal alcohol use), and MR and natural experiments avoid reverse causality (Box 1). In practice, sources of bias varied both across and within each study-type category, as evidenced by our customized RoB tools showing some of the included studies being at higher risk of bias than others. For example, data availability may restrict the extent to which one can test and/or account for potential differential trends in studies exploiting natural experiments such as MLDA. Similarly, data availability may restrict the extent to which one can explore whether (in particular historic) policies affected prenatal alcohol consumption. This is also true for many of the (particularly older) MR studies that did not report genotype associations with maternal alcohol use. Furthermore, ensuring that the analytical strategy identifies effects that are specific to the intrauterine period may be difficult. For example, a reduction in the MLDA in the year of birth is likely to be related to alcohol exposure in that year, but potentially also in the year after. This is less of an issue in studies that exploit temporary changes in alcohol exposure, such as Nilsson, as temporary policies are more likely to only affect alcohol exposure at that point in time only.^42^ In MR studies, one analytical strategy that improves specific attribution of effects to the intrauterine period is using alcohol metabolizing genotypes in the offspring (not just the mothers) as proxy for prenatal alcohol exposure. This is because maternal genotype in theory predisposes to lower or higher alcohol use in pregnancy as well as before and after (therefore it is not specific to the intrauterine period). Additionally, MR studies of intrauterine exposures that do not account for both offspring and maternal genotype can suffer from bias because of violation of the exclusion restriction assumption.^52^ On the other hand, offspring genotype (conditional on maternal genotype) is more specific, since children do not consume alcohol themselves and the only time in early life where they are exposed to alcohol is *in utero*. Therefore, different alcohol metabolizing genotypes in the offspring could modulate prenatal alcohol exposure, independently of maternal alcohol use. This strategy of presenting results for offspring genotype adjusted for maternal genotype was only adopted by a couple of the included MR studies and has the additional advantage of minimizing dynastic effects bias.

An additional strength of some of the natural experiments included here is that they investigated possible mechanisms for the observed effects of prenatal alcohol exposure, in particular through a postulated increase in unplanned pregnancies (also known as ‘compositional changes’). This was explored through, e.g. sensitivity analyses to test whether MLDA changes resulted in more unplanned pregnancies. The idea is that, if MLDA led to an increase in unplanned pregnancies, this may have particularly affected mothers with a systematically different e.g. socio-economic position, whose children also have systematically different outcomes. But these effects are then driven by socio-economic confounding, not (necessarily) only by intrauterine toxicity. This was done by Fertig and Watson^36^ examining the percentage of births recorded with missing paternal information, with the analysis confirming evidence of effect for this in black women, and stronger effects in younger girls (<18 years), thus providing a possible partial explanation for the birthweight effects in their study. Compositional changes or changes in the demographics of mothers giving birth, are also thought to play a role in explaining some of the effect on adverse pregnancy outcomes observed in the study by Zhang^37^ Specifically, since an increase in alcohol taxes appeared to lead to a reduction in pregnancies amongst younger and less educated mothers, who are more likely to experience adverse pregnancy outcomes, maternal age and education (over and above alcohol consumption per se), may explain some of the apparent effect of alcohol. The study by Nilsson^42^ was able to avoid potential bias due to possible compositional changes by focusing on children who were conceived prior to the start of the relaxation of alcohol policy. Hence, his study did not include children who were conceived due to the change in alcohol policy.

Another study by Barreca and Page^39^ additionally investigated the presence of an early selection effect that intrauterine alcohol exposure could have on the least healthy foetuses, by examining gender ratio of live births as a marker of early fetal loss. The authors’ interpretation, although highly speculative, is that this selection indeed is present and could explain the unexpected direction of effect for the main effect analyses in their study.

Small sample sizes in many of the studies (especially for the earlier studies) means that estimates were often imprecise. This was particularly true for the MR studies, some of which were among the first ever to be conducted, and none of which adopted a multi-cohort approach to increase sample size, or multiple genetic variants to improve the variance explained in alcohol consumption, as is recommended and customary in recent times.^53^

Another limitation of the MR and natural experiment studies that were included in this review is the inability to provide dose–response estimates. Instead, they provide estimates of the effect of prenatal alcohol exposure around mean levels of consumption in the study sample. This falls short of the most interesting research question which is whether the effects are linear or whether there is a threshold at low levels of drinking under which alcohol is not harmful to the fetus.

Additionally, for MR studies, only ADH variants have been used and there is a possibility that acetaldehyde is both the deterrent to drinking and the cause of damage, which could lead to null results. Many more loci affecting alcohol intake are now available for future studies,^54^ although their effect on prenatal alcohol use will require validation in studies of pregnant women.

### Strengths and limitations of this systematic review

This systematic review is the first of its kind to explicitly search for and integrate the evidence from different study designs and analytical approaches in a true triangulation framework.^55^ Efforts were made to include studies from different disciplines for the first time, as evidenced by the results of our co-citation analysis (Fig. 2). Alongside this triangulation approach, strengths of this review include the pre-registered protocol http://www.crd.york.ac.uk/PROSPERO/display_record.php? ID=CRD42015015941, and the thorough assessment of RoB through deployment of customized RoB tools.

This literature could be affected by publication bias. Given the lack of sufficient numbers of studies to meta-analyse, we could not investigate publication bias through funnel plots, so it remains speculative whether further unpublished (negative) studies could exist. On the other hand, we notice a trend for a number of studies to attempt replication of certain (positive) seminal papers (e.g. Eilertsen *et al*.^31^ replicating D’Onofrio *et al*.,^30^ Barreca and Page^39^ replicating Fertig and Watson,^36^ Boyles *et al*.^25^ replicating Chevrier *et al*.^24^) and occasionally failing to replicate the original results. Being able to capture these failures to replicate is a strength of the current review.

The main limitation of the review derives from the nature of the evidence we found, the paucity and heterogeneity of which prevented us from pooling effects through meta-analysis. Instead, we systematically grouped results by outcome and study type and examined them for consistency.

Another important limitation is that we cannot infer causal dose–response relationships based on this body of evidence. For example, the effect estimates from MR studies using maternal genotypes effectively refer to the average difference in alcohol to which the offspring are exposed. Therefore, what we can infer is that (often small) increases in prenatal alcohol exposure are associated with lower neurocognitive outcomes and to a lesser extent lower birthweight, based on studies that minimize confounding. The most pressing question of relevance to public health remains whether the recommendation to abstain from alcohol in pregnancy is backed by solid evidence, as opposed to being purely precautionary. We have already explored this extensively with our previous review of observational studies on the effects of low levels of drinking in pregnancy, which concluded that the abstinence advice was mainly a precaution.^6^ The research studies brought together by the current review add to this in a significant way, tipping the balance towards a more solid evidence-base, in particular for neurocognitive and behavioural outcomes.

### Public health/policy and research implications

This review seeks to address an area of great public health impact. Alcohol use in pregnancy is still widespread worldwide,^56^ despite claims that it causes the most common neurodevelopmental impairments, included under the umbrella diagnosis of FASD.^56^ The claims of causality implicit in the diagnostic definition of FASD, however, have occasionally been disputed (e.g. McLennan *et al*.^57^) due to lack of robust evidence of specific alcohol effects on different domains in the child, and whether thresholds apply. This review highlights the need for more studies using a variety of analytical approaches to establish the extent to which prenatal alcohol exposure causes specific neurobehavioral outcomes in the offspring. Studies simultaneously addressing multiple sources of bias are particularly needed (e.g. MR exploiting trio data from fathers, mothers and offspring, to conduct both negative control MR with paternal effects, and analyses accounting for transmitted and un-transmitted alleles,^52^ and 2-samples MR using recently developed approaches to study intrauterine effects),^58^ as are studies allowing for more sophisticated dose–response estimation (e.g. Silverwood *et al*.^59^).

This evidence will then feed into a revised and improved definition of FASD. Results from this review will also inform future reviews of guidelines on alcohol use in pregnancy. The current UK guidelines for example, revised down to abstinence in 2016,^60^ are heavily based on the precautionary principle. The present review of alternative study designs to improve causal inference will strengthen the evidence base for the abstinence recommendation, as well as highlighting the considerable gaps in evidence and quality of studies needed to move the field forward and draw firm conclusions.

## Conclusion

Our understanding of the specific causal effects of alcohol in pregnancy, especially at low levels of exposure, is limited due to biases affecting traditional observational methods and the practical and ethical obstacles to conducting an RCT. Alternative study designs such as MR and natural experiments make an important contribution to our understanding of these effects, as they overcome some of the limitations of traditional methods. Currently, these comprise a modest body of evidence suggestive of a detrimental effect on cognitive outcomes and infant birthweight, which corroborate findings of conventional epidemiological studies. The studies included in this review do not provide evidence on whether the effect of alcohol exposure is linear, or whether there is a safe threshold for drinking in pregnancy, although many of them compare groups of offspring with at most small differences in their prenatal alcohol exposure. Although it remains true that the only way to avoid alcohol-related risks to the fetus is to abstain from alcohol during pregnancy, it is also important to communicate both to mothers-to-be and healthcare professionals that there remains uncertainty in the evidence base for this recommendation,^61^ although we welcome the fact that more and more studies with complementary strengths and weaknesses are emerging in this field.

## Supplementary data

Supplementary data are available at *IJE* online.

## Funding

This work was jointly supported by the National Institute for Health Research Collaboration for Leadership in Applied Health Research and Care West (NIHR CLAHRC West), now recommissioned as NIHR Applied Research Collaboration West (ARC West), the NIHR Bristol Biomedical Research Centre (BRC), and the Medical Research Council (MRC) Integrative Epidemiology Unit. L.M., S.J.L., J.S., H.B.E., V.L., T.J., T.H.M.M. and J.L.D. are fully or partially funded by the NIHR CLAHRC West. The UK MRC (MC_UU_00011/1 and MC_UU_00011/6) and the University of Bristol provide core funding for the MRC Integrative Epidemiology Unit: L.M., D.A.L., G.D.S., A.F. and L.Z. are partially or fully funded through this. A.F., L.Z. and S.vH. are, or were funded, through UK Medical Research Council personal fellowships (Grant refs: MR/M009351/1, G0902144 and G1002345, respectively). D.A.L.’s contribution to this work was supported by the US National Institute of Health (R01 DK10324) and European Research Council (Advanced Grant 669545). D.A.L. (NF-0616–10102) and J.L.D. (NF-SI-0512–10119) are NIHR Senior Investigators. The views expressed here are those of the authors and not necessarily those of the NHS, the MRC, NIHR or the Department of Health and Social Care.

## Supplementary Material

dyz272_Supplementary_Data
