# Supplementary material for: Evidence of detrimental effects of prenatal alcohol exposure on offspring birthweight and neurodevelopment from a systematic review of quasi-experimental studies
Source: Int J Epidemiol. 2020 Jan 29;49(6):1972–95. doi: 10.1093/ije/dyz272 (PMC7825937; doi:10.1093/ije/dyz272)
Supplement: dyz272_Supplementary_Data [file dyz272_supplementary_data.zip › ije-2019-06-0767-File008.docx]

| **Supplementary Table 1.** Search Strategy 1950 to 21·06·2018. MEDLINE, PsycINFO, EMBASE on Ovid; the Cochrane Library including CENTRAL (the Cochrane Central Database of Controlled Trials) on Wiley Interscience; and Science Citation Index, Social Science Citation Index, on Web of Science. | |  |  |  |
| --- | --- | --- | --- | --- |
| 1 exp pregnancy/ (719661) | |  |  |  |
| 2 Pregnant Women/ (5199) | |  |  |  |
| 3 preconception care/ or prenatal care/ (21644) | |  |  |  |
| 4 exp "embryonic and fetal development"/ (212127) | |  |  |  |
| 5 Fetus/ (68424) |  |  |  |  |
| 6 exp Pregnancy Complications/ (343999) | |  |  |  |
| 7 (prepregnan$ or conception or preconception or pregnan$ or prenatal$ or pre-natal$ or  f?etal or f?etus  or in utero).ti,ab. (588557) | | | |  |
| 8 Maternal exposure/ (5534) | |  |  |  |
| 9 or/1-8 (1079283) |  |  |  |  |
| 10 Ethanol/ (73449) |  |  |  |  |
| 11 Alcohol dehydrogenase/ (5809) | |  |  |  |
| 12 Aldehyde Oxidoreductases/ (3672) | |  |  |  |
| 13 exp Drinking Behavior/ (57821) | |  |  |  |
| 14 Temperance/ (2430) |  |  |  |  |
| 15 alcohol$.ti. (106954) |  |  |  |  |
| 16 (alcohol dehydrogenase or acetaldehyde dehydogenase).ti,ab. (8557) | |  |  |  |
| 17 ((alcohol or alcoholic) adj3 (drink$ or exposure or consumption or consume$ or consuming  or low or light or moderat$ or abstin$ or abstain$)).ti,ab. (49470) | | | | |
| 18 ((low or light or moderate or abstin$ or abstain$ or pattern$ or behavio?r$) adj3 drink$).ti,ab.  (10558) | | |  |  |
| 19 (ADH1B$ or teetotal$ or temperance or nondrink$ or non-drink$).ti,ab. (3093) | |  |  |  |
| 20 Genome-Wide Association Study/ or Linkage Disequilibrium/ or genotype/ or phenotype/ or  polymorphism, genetic/ or (polymorphism$ or ((gene or genes or genetic or genotyp$) adj3  (instrument$ or variant$ or variable$ or variability or variabilities or variance$))).ti,ab. (494904) | | | | |
| 21 *Alcohols/ or exp *Alcohol-Related Disorders/ or (alcohol adj3 (misuse or "use" or abuse or  addict$ or dependence or response$ or susceptibility)).ti,ab. (110843) | | | | |
| 22 20 and 21 (3108) |  |  |  |  |
| 23 or/10-19,22 (213050) |  |  |  |  |
| 24 9 and 23 (11403) |  |  |  |  |
| 25 letter/ (861500) |  |  |  |  |
| 26 editorial/ (368458) |  |  |  |  |
| 27 news/ (166329) |  |  |  |  |
| 28 exp historical article/ (325965) | |  |  |  |
| 29 Anecdotes as topic/ (4586) | |  |  |  |
| 30 comment/ (610211) |  |  |  |  |
| 31 case report/ (1708277) | |  |  |  |
| 32 (letter or comment$).ti. (100442) | |  |  |  |
| 33 or/25-32 (3417468) |  |  |  |  |
| 34 randomized controlled trial/ or Randomized Controlled Trials as Topic/ or random$.ti,ab. (888907) | | |  |  |
| 35 33 not 34 (3386180) |  |  |  |  |
| 36 animals/ not humans/ (3889478) | |  |  |  |
| 37 exp Animals, Laboratory/ (730783) | |  |  |  |
| 38 exp Animal Experimentation/ (6477) | |  |  |  |
| 39 exp Models, Animal/ not humans/ (310115) | |  |  |  |
| 40 exp rodentia/ (2688128) | |  |  |  |
| 41 (rat or rats or mouse or mice).ti. (1123907) | |  |  |  |
| 42 or/35-41 (7849766) |  |  |  |  |
| 43 24 not 42 (6498) |  |  |  |  |
| 44 meta-analysis/ (52850) | |  |  |  |
| 45 meta-analysis as topic/ (13933) | |  |  |  |
| 46 (meta analy$ or metaanaly$ or metanaly$ or meta regression).ti,ab. (72390) | |  |  |  |
| 47 ((systematic$ or evidence$ or realist or narrative or literature) adj2 (review$ or overview$)).ti,ab.  (171877) | | |  |  |
| 48 "review of reviews".ti,ab. (211) | |  |  |  |
| 49 (reference list$ or bibliograph$ or hand search$ or manual search$ or relevant journals).ab.  (27094) | | |  |  |
| 50 (search strategy or search criteria or systematic search or study selection or data extraction).ab.  (28877) | | |  |  |
| 51 (search$ adj4 literature).ab. (30686) | |  |  |  |
| 52 (medline or pubmed or cochrane or embase or psychlit or psyclit or psychinfo or cinahl or science  Citation index or bids or cancerlit).ab. (95294) | | | | |
| 53 cochrane.jw. (11053) |  |  |  |  |
| 54 ((multiple treatment$ or indirect or mixed) adj2 comparison).ti,ab. (991) | |  |  |  |
| 55 or/44-54 (293491) |  |  |  |  |
| 56 43 and 55 (201) |  |  |  |  |
| 57 epidemiologic studies/ (6077) | |  |  |  |
| 58 ep.fs. (1223653) |  |  |  |  |
| 59 exp case control studies/ (691899) | |  |  |  |
| 60 exp cohort studies/ (1394149) | |  |  |  |
| 61 cross-sectional studies/ (185407) | |  |  |  |
| 62 Mendelian Randomization Analysis/ (229) | |  |  |  |
| 63 (case control or negative control).ti,ab. (93097) | |  |  |  |
| 64 (cohort adj (study or studies or analys$)).ti,ab. (98537) | |  |  |  |
| 65 ((follow up or observational) adj (study or studies)).ti,ab. (87771) | |  |  |  |
| 66 ((longitudinal$ or retrospectiv$ or prospectiv$) and (study or studies or studied or review$ or  analys$ or cohort$)).ti,ab. (870699) | | | | |
| 67 cross sectional.ti,ab. (183479) | |  |  |  |
| 68 (mendel$ or natural experiment$).ti,ab. (11082) | |  |  |  |
| 69 ((family or sibling or population) adj3 (study or studies)).ti,ab. (115245) | |  |  |  |
| 70 or/57-69 (2937160) |  |  |  |  |
| 71 exp guideline/ (25834) | |  |  |  |
| 72 guidelines as topic/ or practice guidelines as topic/ (114690) | |  |  |  |
| 73 guideline$.mp. (291621) | |  |  |  |
| 74 or/71-73 (291621) |  |  |  |  |
| 75 55 or 70 or 74 (3362878) | |  |  |  |
| 76 43 and 75 (3349) |  |  |  |  |
| 77 limit 76 to english language (3096) | |  |  |  |
| 78 ((smok$ or drug$ or tobacco or nicotine or cigarette$ or substance$ or methamphetamine$  or amphetamine$ or cocaine$ or heroin or cannabis or marijuana) not (alcohol or alcoholic or drink$)).ti.  (489662) | | | | |
| 79 77 not 78 (2822) |  |  |  |  |
