# Supplementary material for: Evidence of detrimental effects of prenatal alcohol exposure on offspring birthweight and neurodevelopment from a systematic review of quasi-experimental studies
Source: Int J Epidemiol. 2020 Jan 29;49(6):1972–95. doi: 10.1093/ije/dyz272 (PMC7825937; doi:10.1093/ije/dyz272)
Supplement: dyz272_Supplementary_Data [file dyz272_supplementary_data.zip › ije-2019-06-0767-File009.pdf]

Supplementary Table 2.

Study (year)	Outcomes	Additional results
[30] Fertig (2009)	Low birth weight (<2500g)	White women:
		MLDA of 18 main effect; -0.10% (SE 0.04)[3]
		MLDA of 18 x mother ≤ 17 years of age interaction; 0.24% (SE 0.13)[3]
		MLDA of 18 x mother 18-20 years of age interaction; 0.13% (SE 0.06)[3]
		Black women:
		MLDA of 18 main effect; 0.45% (SE 0.17)[3]
		MLDA of 18 x mother ≤ 17 years of age interaction; 1.01% (SE 0.24)[3]
		MLDA of 18 x mother 18-20 years of age interaction; 0.60% (SE 0.15)[3]
		White women:
		MLDA of 18 main effect; -0.18% (SE 0.07)[3]
		MLDA of 18 x mother ≤ 17 years of age interaction; 0.43% (SE 0.16)[3]
	Preterm birth (<37 weeks gestation)	MLDA of 18 x mother 18-20 years of age interaction; 0.06% (SE 0.08)[3]
		Black women:
		MLDA of 18 main effect; -0.80% (SE 0.21)[3]
		MLDA of 18 x mother ≤ 17 years of age interaction; 1.32% (SE 0.28)[3]
		MLDA of 18 x mother 18-20 years of age interaction; 0.68% (SE 0.14)[3]
		White women:
		MLDA of 18 main effect; -0.08% (SE 0.07)[3]
		MLDA of 18 x mother ≤ 17 years of age interaction; -0.03% (SE 0.04)[3]
	Congenital anomalies	MLDA of 18 x mother 18-20 years of age interaction; -0.02% (SE 0.02)[3]
		Black women:
		MLDA of 18 main effect; -0.39% (SE 0.26)[3]
		MLDA of 18 x mother ≤ 17 years of age interaction; -0.02% (SE 0.07)[3]
		MLDA of 18 x mother 18-20 years of age interaction; -0.04% (SE 0.03)[3]
[14] Zhang (2011)	Low birthweight (<2,500 grams)	White women:
		MLDA of 18; no change
		MLDA of 19; no change
		MLDA of 20; no change
		MLDA of 21; no change
		Black women:
		MLDA of 18; 0.18% (p< 0.001)[5]
		MLDA of 19; 0.20% (p <0.07)[5]
		MLDA of 20; -0.07% (p=0.267)[5]
		MLDA of 21; -0.28% (p<0.001)[5]
	Low APGAR scores (<7)	White women:
		MLDA of 18; 0.36% (p< 0.001)[5]
		MLDA of 19; -0.50% (p=0.003)[5]
		MLDA of 20; -0.40% (p=0.002)[5]
		MLDA of 21; -0.61% (p<0.001)[5]
		Black women:
		MLDA of 18; 3.72% (p< 0.001)[5]
		MLDA of 19; -2.57% (p<0.001)[5]
		MLDA of 20; -3.68% (p<0.001)[5]
		MLDA of 21; -4.98% (p<0.001)[5]
	Pre-maturity birth (< 37 weeks'	White women:
	gestation)	MLDA of 18; no change
		MLDA of 19; no change
		MLDA of 20; no change
		MLDA of 21; no change
		Black women:
		MLDA of 18; 0.61% (p< 0.10)[5]
		MLDA of 19; -0.56% (p=0.210)[5]
		MLDA of 20; -0.27% (p=0.502)[5]
		MLDA of 21; -0.95% (p=0.075)[5]
[31] Barreca	Birthweight (<2500g)	White women:
(2013)		MLDA of 18 main effect; -0.12% (SE 0.07)[4]
		MLDA of 18 x mother 14- 17 years of age interaction; -0.16% (SE 0.10)[4]
		MLDA of 18 x mother 18-20 years of age interaction; 0.12% (SE 0.05)[4]
		Mean of outcome: 6.1
		Black women:

		MLDA of 18 main effect; -0.18% (SE 0.16)[3]<br>MLDA of 18 x mother 14- 17 years of age interaction; -0.07% (SE 0.15)[4]<br>MLDA of 18 x mother 18-20 years of age interaction; -0.20% (SE 0.13)[4]<br>Mean of outcome:12.9
	Gestation (<37 weeks)	White women:<br>MLDA of 18 main effect; 0.08% (SE 0.10)[4]<br>MLDA of 18 x mother 14- 17 years of age interaction; -0.06% (SE 0.09)[4]<br>MLDA of 18 x mother 18-20 years of age interaction; 0.05% (SE 0.09)[4]<br>Mean of outcome: 8.7<br>Black women:<br>MLDA of 18 main effect; no change<br>MLDA of 18 x mother 14- 17 years of age interaction; -0.15% (SE 0.24)[4]<br>MLDA of 18 x mother 18-20 years of age interaction; -0.33% (SE 0.16)[4]<br>Mean of outcome: 18.2
	Apgar score (<7)	White women:<br>MLDA of 18 main effect; 0.43% (SE 0.34)[4]<br>MLDA of 18 x mother 14- 17 years of age interaction; 0.34% (SE 0.46)[4]<br>MLDA of 18 x mother 18-20 years of age interaction; 0.009% (SE 0.24)[4]<br>Mean of outcome: 904.2<br>Black women:<br>MLDA of 18 main effect; 0.21% (SE 0.74)[4]<br>MLDA of 18 x mother 14- 17 years of age interaction; 0.10% (SE 0.75)[4]<br>MLDA of 18 x mother 18-20 years of age interaction; 0.58% (SE 0.48)[4]<br>Mean of outcome: 891.9
	Congenital anomaly	White women:<br>MLDA of 18 main effect; -0.23% (SE 0.16)[4]<br>MLDA of 18 x mother 14- 17 years of age interaction; 0.28% (SE 0.06)[4]<br>MLDA of 18 x mother 18-20 years of age interaction; 0.05% (SE 0.03)[4]<br>Mean of outcome: 7.9<br>Black women:<br>MLDA of 18 main effect; -0.43% (SE 0.37)[4]<br>MLDA of 18 x mother 14- 17 years of age interaction; -0.38% (SE 0.10)[4]<br>MLDA of 18 x mother 18-20 years of age interaction; 0.03% (SE 0.57)[4]<br>Mean of outcome: 8.6
	Female	White women:<br>MLDA of 18 main effect; 0.007% (SE 0.12)[4]<br>MLDA of 18 x mother 14- 17 years of age interaction; -0.12% (SE 0.12)[4]<br>MLDA of 18 x mother 18-20 years of age interaction; 0.063% (SE 0.10)[4]<br>Mean of outcome: 48.6<br>Black women:<br>MLDA of 18 main effect; -0.06% (SE 0.22)[4]<br>MLDA of 18 x mother 14- 17 years of age interaction; -0.21% (SE 0.20)[4]<br>MLDA of 18 x mother 18-20 years of age interaction; 0.46% (SE 0.20)[4]<br>Mean of outcome: 49.2

Abbreviations: LBW: Low birth weight, APGAR: Appearance, Pulse, Grimace, Activity, Respiration, MLDA: minimum legal drinking age, OR: Odds ratio, CI: Confidence intervals, MD: Mean difference, SD: standard deviation; SGA: Small for gestational age, RR: relative risk, Numbers in bold: $P < 0.05$

[1]Adjustments: Sex, other parent's alcohol consumption, maternal age, parity, socio-economic position, ethnicity, and, maternal and paternal education and smoking

[2]Adjustments: Sex, social class, parity, ethnicity, house ownership, crowding, maternal and paternal education and smoking, and, paternal effects in the maternal model and maternal effects in the paternal model.

[3]Adjustments: State fixed effects, year–month fixed effects, maternal age fixed effects, state-specific time trends, and birth characteristic controls.

[4] Adjustments: State fixed effects, year-by-month fixed effects, age fixed effects, state-specific trends, age-by-year fixed effects, state-by-age fixed effects, and state-by-year fixed effects.

[5] Adjustments: State fixed effects, year fixed effects, mother's education, age, marital status, smoking during pregnancy, real income per capita, and real beer taxes (federal plus state level)
