# Supplementary material for: Evidence of detrimental effects of prenatal alcohol exposure on offspring birthweight and neurodevelopment from a systematic review of quasi-experimental studies
Source: Int J Epidemiol. 2020 Jan 29;49(6):1972–95. doi: 10.1093/ije/dyz272 (PMC7825937; doi:10.1093/ije/dyz272)
Supplement: dyz272_Supplementary_Data [file dyz272_supplementary_data.zip › ije-2019-06-0767-File010.docx]

**Risk of bias assessment for MR studies**

Review of alternative designs for alcohol in pregnancy for any child health and education outcomes

Assessor ID:

Study ID:

| **Bias domain** | **Question** | **High** | **Moderate** | **Low** | **Judgement**  **Low risk, some concerns, high risk** |
| --- | --- | --- | --- | --- | --- |
| **Weak instrument bias** | Strength of association between instrument and exposure *F* statistic < 10 *in the same sample*  (< 10 indicating a weak instrument) | F<10 | F= missing or F~10 | F>>10 |  |
| **Genetic confounding bias** | Reported test on association between confounders and IV  (testing the assumption that the instrument is associated with your outcome only via your exposure) | Yes AND there is an obvious association | Not presented or  Yes presented AND there is some degree of association | Presented and no obvious association |  |
| **‘Other’ Confounding bias** | Included confounders in the IV analysis  yes= may introduce bias *except for ethnicity* | Yes (lifestyle factors) |  | no |  |
| **Additional direct effects between IV and outcome (exclusion restriction assumption)** | Presence of pleiotropy for genetic IVs | genetic IVs with no knowledge of mechanism for G-alcohol association (e.g. GWAS hit, could be acting through any pathway…) | Biologically plausible alcohol-specific mechanism of association for G-alcohol (e.g. alcohol metabolising genetic variants) | same as moderate AND checks that there is no other known effect of genetic variants on outcome or its risk-factors, which is likely independent of alcohol |  |
| **Bias due to selection of participants** | Homogenous population or similar ancestry  **If no**  Stratified by ethnicity or adjusted for population stratification **(yes/no)** | non-homogenous population (e.g. black and white together, etc.) | population described as homogenous (e.g. whites only) BUT not corrected for ancestry informative markers like principal components derived from GWAS | population described as homogenous (e.g. whites only) AND corrected for ancestry informative markers like principal components derived from GWAS |  |

Study outcomes:
