# Supplementary material for: Evidence of detrimental effects of prenatal alcohol exposure on offspring birthweight and neurodevelopment from a systematic review of quasi-experimental studies
Source: Int J Epidemiol. 2020 Jan 29;49(6):1972–95. doi: 10.1093/ije/dyz272 (PMC7825937; doi:10.1093/ije/dyz272)
Supplement: dyz272_Supplementary_Data [file dyz272_supplementary_data.zip › ije-2019-06-0767-File011.docx]

**Risk of bias assessment for Parental Comparison studies (note- in this study design, the estimate of interest isn’t just the estimate of effect of maternal exposure on offspring outcome, but rather the comparison of the difference in effect sizes between maternal and paternal effect estimates).**

Review of alternative designs for alcohol in pregnancy for any child health and education outcomes

Assessor ID:

Study ID: Study outcomes:

| **Bias domain** | **Question** | **High** | **Moderate** | **Low** | **Mark** |
| --- | --- | --- | --- | --- | --- |
| **Confounding bias** | Assumption that paternal exposures share same confounding structure as maternal exposure – both prenatal and postnatal exposures (if outcome is postnatal)  *papers should show distribution of measured confounders for paternal and maternal exposure* | not shown or very dissimilar at least for key confounders (defined in our paper 1, pls list here) | somewhat dissimilar for some of the main confounders | Similar for all main confounders |  |
|  | Have exposures been measured in both parents at same time in pregnancy? | No. paternal exposure measured at very different time (eg before pregnancy or in last trimester) compared to maternal exposure (eg in first trimester) | Not exactly, but almost (eg within 3 months of each other…) | Yes, measured exactly at the same time during pregnancy. |  |
|  | Has nonpaternity been taken into account for phenotypic traits? | nothing done AND population with estimated high rates of non-paternity | nothing done BUT low estimated rates of non-paternity | sensitivity analyses run for various degrees of nonpaternity |  |
|  | likelihood of a paternal effect also present? (based on previous epidemiological literature and/or mechanisms – eg paternal smoking could affect the offspring)  (If parental associations are of similar magnitude, could this be due to paternal pathways rather than shared confounding?) | yes | Unsure/unknown | no |  |
| **Assessment bias** | Exposure measure e.g. recall bias. This could affect mothers more than fathers as the latter are unlikely to hold their own drinking behaviour (during their partner’s pregnancy) responsible for their offspring’s outcomes. | Retrospective measurement (after the outcome is known) |  | Prospective measurement (during pregnancy) |  |
