# Supplementary material for: Evidence of detrimental effects of prenatal alcohol exposure on offspring birthweight and neurodevelopment from a systematic review of quasi-experimental studies
Source: Int J Epidemiol. 2020 Jan 29;49(6):1972–95. doi: 10.1093/ije/dyz272 (PMC7825937; doi:10.1093/ije/dyz272)
Supplement: dyz272_Supplementary_Data [file dyz272_supplementary_data.zip › ije-2019-06-0767-File012.docx]

**Risk of bias assessment for Sibling Comparison Studies**

Review of alternative designs for alcohol in pregnancy for any child health and education outcomes

Assessor ID:

Study ID:

Study outcomes:

| **Bias domain** | **Question** | **High** | **Moderate** | **Low** | **Mark** |
| --- | --- | --- | --- | --- | --- |
| **Confounding bias** | Birth order | Exposed siblings share the same birth order | Same as high BUT study adjusts for other factors during and after pregnancy such as smoking behaviour | Birth order balanced OR  birth order taken into account in analyses |  |
|  | Individual level confounding | Did not correct for any intrauterine specific exposures or postnatal confounders or any individual level confounders | Corrected for at least intrauterine exposures BUT not for postnatal factors | Corrected for several confounders |  |
| **Assessment bias** | Exposure measure e.g. recall bias | Retrospective measurement (after the outcome is known) |  | Prospective measurement |  |
|  | Was a robust outcome measure used  Outcome measured objectively (blind of the exposure) | No (e.g. Parental reporting) |  | Yes (e.g. routine data) |  |
| **Selection bias** | Was there likely loss to follow up bias for a cohort study | Yes |  | No |  |
