# Supplementary material for: Evidence of detrimental effects of prenatal alcohol exposure on offspring birthweight and neurodevelopment from a systematic review of quasi-experimental studies
Source: Int J Epidemiol. 2020 Jan 29;49(6):1972–95. doi: 10.1093/ije/dyz272 (PMC7825937; doi:10.1093/ije/dyz272)
Supplement: dyz272_Supplementary_Data [file dyz272_supplementary_data.zip › ije-2019-06-0767-File013.docx]

**Risk of bias assessment for Natural experiment studies (note – the natural experiment is used as an instrumental variable either formally or by inference)**

Review of alternative designs for alcohol in pregnancy for any child health and education outcomes

Assessor ID:

Study ID:

| **Bias domain** | **Question** | **High** | **Some Risk** | **Low** | **Mark** |
| --- | --- | --- | --- | --- | --- |
| **Confounding 1** | Were the populations being compared similar with the exception of the naturally randomized exposure? (usually checked by examining potentially confounding characteristics in the different populations) | No (eg different ethnicities, deprivation levels, age distribution, etc…) | Not clear, no data | Yes |  |
| **Confounding 2** | Has the outcome been changing over time differentially in the populations with and without the naturally randomized exposure? (this is usually checked by examining longer term time trends including observations BEFORE the change/natural experiment takes place) | Yes – eg evidence of differential trends btw natural experiment and comparison populations | No data (newly reported or cited) on trends | No differential trends |  |
| **Confounding 3** | Have there been any other state-level changes coinciding with the natural experiment/intervention? (e.g. change in availability of tobacco or other drugs, major changes in primary or secondary healthcare etc in the naturally randomized exposure population but not the control population) | Yes AND only double differences (differences in differences) estimates available | No data or comment in the discussion | No, OR the effect was estimated through ‘triple differences’ which accounts for any State-level differences (eg assuming the ‘alcohol’ change affects 18-21 yr old mums only, and the other change affects older women too, then if older women in the same State are part of the comparison (like is in triple difference estimates), then only alcohol-specific effects applied to the 18-21 age group) |  |
| **Instrument strength** | Strength of adherence to law change (natural experiment) or other measures of compliance with instrument (eg evidence of more drinking in 18-21 year old women if the minimum legal drinking age is lowered from 21 to 18, etc…) | Low adherence/instrument strength (although this could also result in under-estimation of the effect size) | no method of assessing this (no data reported or cited) | High adherence/instrument strength |  |
| **Assessment** | Have identical methods for measuring the outcome been used in each population (exposed and unexposed)? | No |  | Yes |  |
| **Selection bias** | Did the intervention/natural experiment cause a change in the distribution/characteristics of women getting pregnant, such to introduce selection bias? (eg if by lowering minimum legal drinking age a lot more young women get pregnant accidentally, which might result in poorer perinatal outcomes etc due to who these women are, more than to the prenatal alcohol exposure per se…) | Yes ( eg pregnancies conceived during the natural experiment period were included AND results of sensitivity analyses exploring the demographics of these pregnancies showed large differences such as higher than expected rates of missing paternal information suggesting ‘accidental’ pregnancies..) |  | No (eg all pregnancies conceived during the natural experiment period were excluded, only those conceived before the start of the period but going on during the period and hence at different risk of being exposed to alcohol, were included, OR sensitivity analyses suggested low likelihood of different demographics in women conceiving during the natural experiment…) |  |

Study outcomes:
